# Minimally invasive versus mini-open transforaminal lumbar interbody fusion in managing low-grade degenerative spondylolisthesis

**DOI:** 10.1007/s00701-024-06231-7

**Published:** 2024-09-12

**Authors:** Elsayed Mohamed Selim Ali, Mohamed Abdeen, Mohammed Khalid Saleh

**Affiliations:** Orthopedic Department, Faculty of Medicine, Zagazig University Hospital, Zagazig, Egypt

**Keywords:** MIS-TLIF, Mini-open TLIF, Degenerative spondylolisthesis, Minimally invasive, Mini-open and sublaminar laminoplasty

## Abstract

**Data background:**

Because the traditional open-TLIF approach has several drawbacks, minimally invasive surgery (MIS) approaches for TLIF (MISTLIF) have been developed to speed up recovery after surgery and minimize pressure on the para-spinal muscles, necessitating a cost-utility analysis for comparison in healthcare reforms.

**Objectives and aim of the work:**

This study aimed to compare the radiological and clinical parameters between mini-open TLIF and minimally invasive transforaminal lumbar interbody fusion (MIS-TLIF) surgery in patients with single-level lumbar degenerative spondylolisthesis.

**Hypothesis:**

This study hypothesizes that both minimally invasive and mini-open methods using sublaminar trimming laminoplasty (SLTL) (while preserving midline structures) and interbody cages have comparable mid- and long-term clinical and radiological outcomes.

**Methods:**

Retrospective analyses were performed on 120 patients who underwent single-level TLIF procedures with a minimum of two years of follow-up utilizing either the mini-open (n = 60) or MIS (n = 60) technique. Records of the operation's time frame, intraoperative fluoroscopy, blood loss, postoperative drainage volume, duration of bed rest, and complications were recorded. The Oswestry Disability Index (ODI) and visual analog scale (VAS) scores for both groups were utilized to assess improvements in clinical scores, and t tests were employed to statistically compare the outcomes. For comparison, radiological parameters, including lumbar lordosis, pelvic incidence (PI), and localized lordosis at the index level, were measured preoperatively, postoperatively, and at the final follow-up. To assess postoperative interbody fusion, the Bridwell grading system was used.

**Results:**

In the Mini-open TLIF group, the average follow-up time was 24.91 ± 5.7 months, while in the MIS-TLIF group, the average follow-up time was 25.15 ± 4.2 months. In the MIS-TLIF group, the mean operation and radiological time were longer. However, compared to the Mini-open TLIF group, the MISTLIF group experienced less blood loss and a shorter hospital stay. The MIS-TLIF group outperformed the Open-TLIF group in terms of the VAS score for back pain and the ODI at less than 6 months following surgery, and the differences were statistically significant. However, at the final follow-up, there were no statistically significant differences in the VAS score for the back between the two groups, but the ODI score was significantly greater in the MIS-TLIF group. Both groups' lumbar lordosis and focal lordosis significantly improved at the index level, with the Mini-open-TLIF group showing more focal lordosis. The interbody fusion rate did not significantly differ between the two groups.

**Conclusion:**

MIS-TLIF and mini-open-TLIF can be surgically effective in treating single-level degenerative lumbar spine spondylolisthesis.

## Introduction

Patients with degenerative spondylolisthesis of the lumbar spine usually present with radicular leg pain or neurogenic claudication, with or without low back pain. Whenever conservative management fails, patients are offered surgery. In the case of stable spondylolisthesis documented on dynamic radiographs, patients can be treated with decompression alone, and the modest difference in favor of additional instrumentation does not justify the associated higher costs for implants and longer duration of surgery [5]. Most patients with unstable spondylolisthesis are treated with nerve root decompression in addition to pedicle screw fixation and interbody fusion.

Minimally invasive spine surgery (MISS) is becoming increasingly popular worldwide. The rationale behind minimally invasive techniques is less tissue damage, reduced back pain leading to a shorter rehabilitation period, and faster return to work and resumption of daily activities [1, 11].

In 2014, Wulu et al. described his new sublaminar trimming laminoplasty technique. This technique comprises aspects of laminotomy and laminectomy. It is proposed to remove tissue around the thecal sac and nerve root to widen the spinal canal while preserving structures that stabilize the spine, such as the facet joint, interspinous ligament, and supraspinous ligament [12]. In this study, we used this technique for mini-open access for interbody fusion to benefit both decompression and interbody fusion together with posterior and posterolateral fusion while preserving midline structures.

This study hypothesizes that both minimally invasive and mini-open methods using sublaminar trimming laminoplasty (SLTL) (while preserving midline structures) and interbody cages have comparable mid- and long-term clinical and radiological outcomes.

## Patients and methods

The primary outcomes will be the VAS score for leg pain and back pain, and the 2nd outcome will be the ODI score, return to work, blood loss, blood transfusion, length of hospital stay, incidence of reoperation, and documentation of fusion. A retrospective study was performed using the medical records of our University Hospital for patients suffering from single-level lumbar low-grade degenerative spondylolisthesis who underwent surgical intervention with minimally invasive surgery (group 1, sixty patients) or mini-open surgery (group 2, sixty patients). Patients younger than 18 years, patients diagnosed with lytic and/or high-grade spondylolisthesis, patients who presented with infection, tumors, trauma, multiple levels of degenerative spondylolisthesis, and morbid obesity with a body mass index (BMI) greater than 40 were excluded.

On CT slices taken every six months until fusion, the modified Bridwell criteria for fusion of the lumbar spine were used. A sufficient fusion rate was determined using both grades I and II.

## Surgical techniques

### Group I

#### Minimally invasive interbody fusion with percutaneous pedicle screw insertion

*MIS‑TLIF procedure.* After successful general anesthesia, the patient was placed in a prone position with chest and hip pads to avoid abdominal compression. Before the operation, a C-arm machine was used to fluoroscopically locate the surgical segment and mark the pedicle. After routine disinfection and towel laying, we made a bilateral longitudinal incision of approximately 2.5 cm at the marked position (Wiltse approach) and cut the skin, subcutaneous tissue and deep fascia in sequence. The four guide wires for the percutaneous pedicular screws were inserted before decompression was performed, and then the muscles were bluntly separated using tubular dilators to reach our docking site at the facet joint until insertion of the working channel. After fluoroscopy, the operation segment was correct, part or all of the upper and lower articular processes were removed according to the patient’s symptoms and spinal canal anatomy, and part of the lamina was removed to enlarge the spinal canal.

**Fig. 1:**
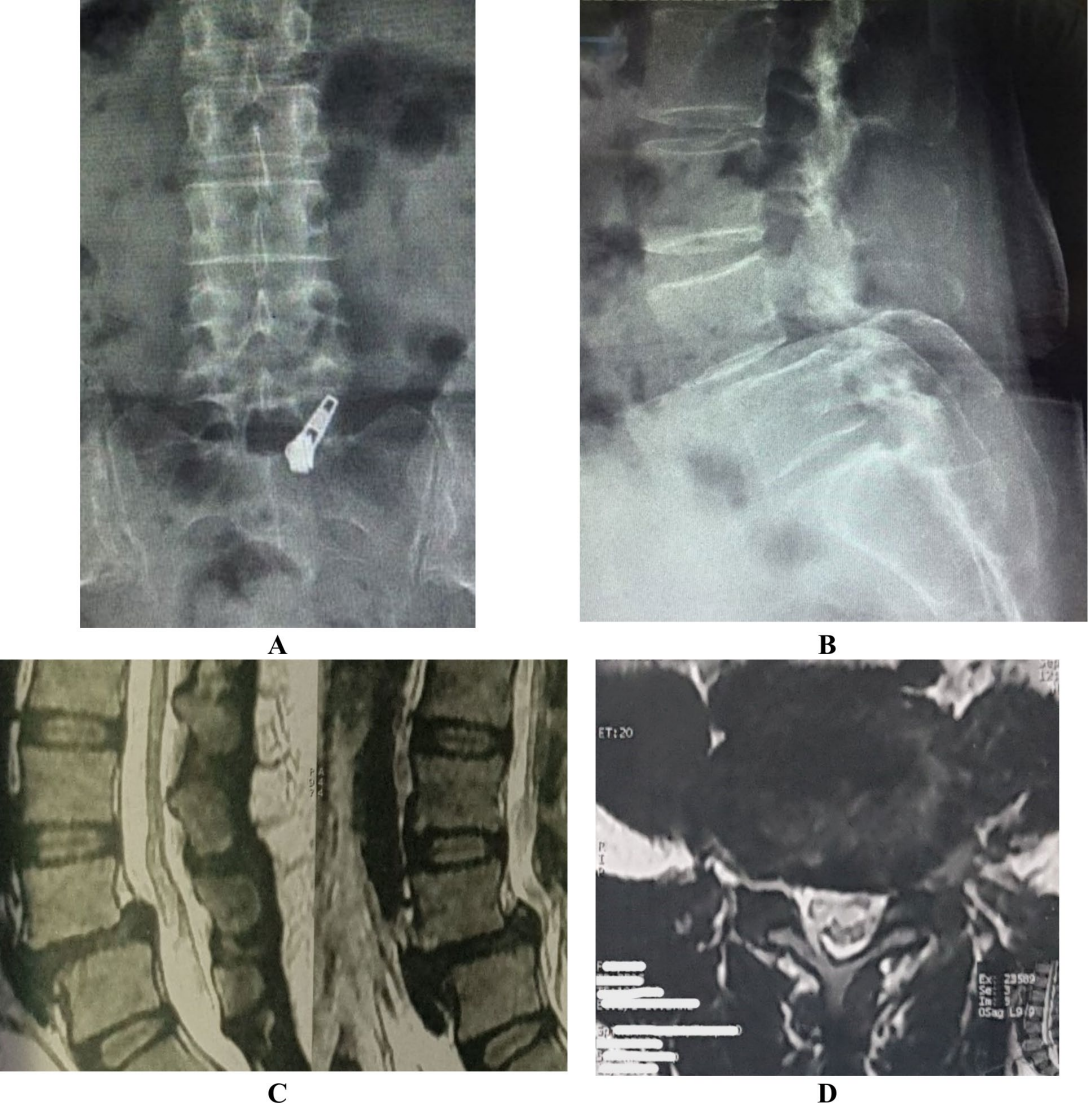
MISTLIF case**: (A-B)** show antero-posterior and lateral radiology of the slipped level and **(C-D)** show the MRI photo of that level (sagittal and axial cut)

**Fig. 2:**
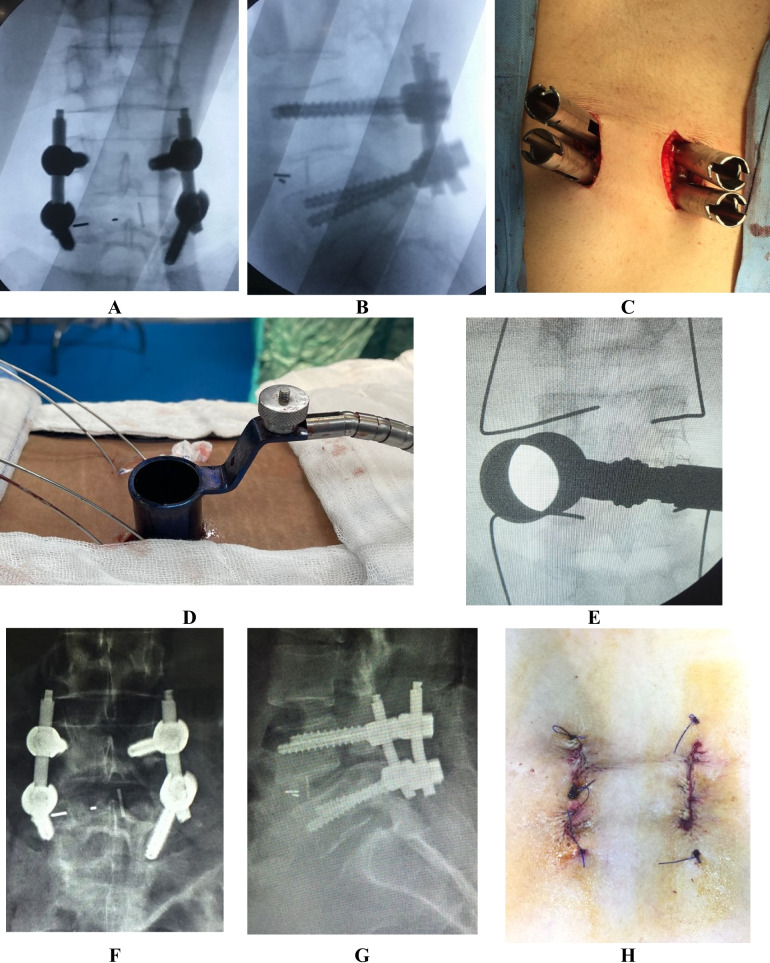
MISTLIF case**: (A–B)** show intraoperative fluoroscopic image antro-posterior and lateral view of the slipped level after insertion of the screws and rods, **(C)** show intraoperative photo of the wound, and (**D**) the tubular system in place, (**E**) C-Arm photo of the tubular system fixed on the facet **(F-G)** postoperative X-ray intro-posterior and lateral view of the slipped level and **(H)** shows the wound photo after closure of the wound

We decompressed the dural sac, the traversing nerve roots and the exiting nerve roots. The nucleus pulposus was removed from the degenerated nucleus, the endplate cartilage was scraped off, and the autogenous bone and BMP were implanted and beaten tightly. After a successful mold trial, a suitable PEEK cage was placed into the center of the intervertebral space through the Kambin triangle area. Percutaneous pediclular screws were placed over the guide wires previously inserted, the rods were inserted manually, and final tightening was performed after reduction of the spondylolisthesis (Fig. 1 and 2**)**.

### Group II

#### Mini-open sublaminar trimming laminoplasty with interbody cage insertion

**Fig. 3:**
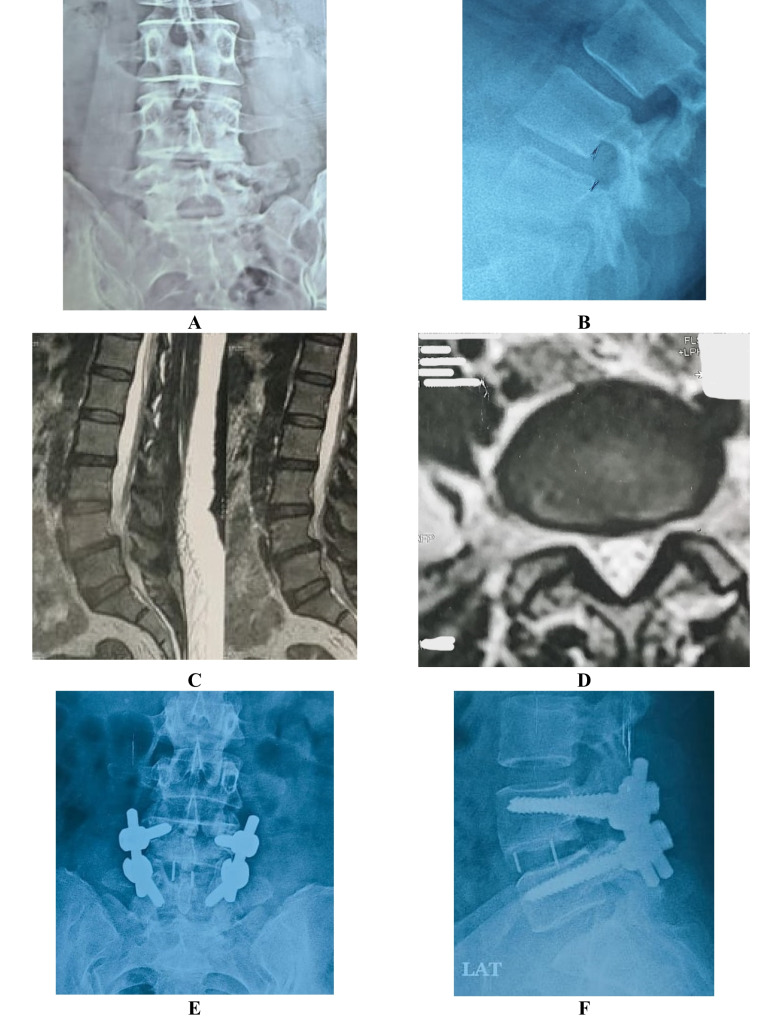
Mini-open TLIF case: **(A–B)** antero-posterior and lateral radiology of the slipped level and **(C-D)** show the sagittal and axial cut of MRI photo of that level **(E–F)** postoperative X-ray intro-posterior and lateral view of the slipped level

**Fig. 4:**
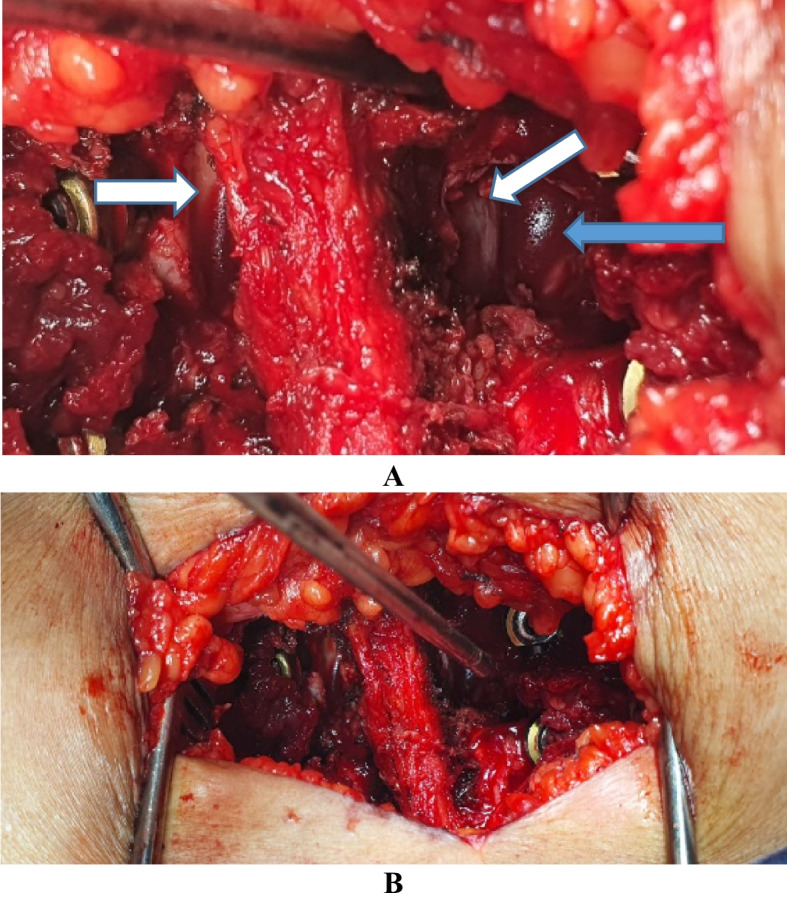
Intraoperative photo of the Mini-open TLIF case with the spinous process in the midline and the lateral edge of the Dural sac seen bilateral (two white arrows) after performing the decompression and the blue arrow points the site of cage entry (**A**), (**B**) a pointer marking the window for cage entry

In this technique, a posterior midline incision approximately 3 cm centered over the affected level is made. We preserved midline structures, including the spinous process and the inter-spinous ligament. The lower part of the laminae of the level above was removed together with the upper part of the laminae of the lower level to complete the foraminotomy with removal of the superior articulating process of the lower level. The ligamentum flavum was removed with a curved curette. The pedicular screws were inserted through the prepared holes together with the rods, and the screws were finally tightened. The interbody cage was inserted after the intervertebral disc was removed and the bed was prepared. Decortication of the posterior structures with insertion of the posterolateral graft (Fig. 3 and 4**)**.

## Statistical analysis

Data were coded, entered and analyzed using SPSS (Statistical Package for Social Science) version 25. Qualitative data were presented as frequencies and percentages while, quantitative data were presented as mean, standard deviations. Comparison of quantitative normally distributed data of two independent groups was done using Student t test, while comparison of quantitative dependent normally distributed data with repeated measures over different period of time was done using Repeated Measures ANOVA. Comparisons among qualitative variables was done using Chi Square test. P value (≤ 0.05) was considered statistically significant difference and P value < 0.001 was considered high statistically significant difference.

## Results

The MIS-TLIF group included 34 males and 26 females, with an average age of 40.4 ± 13.6 years. Of those, 45 had grade 1 degenerative spondylolisthesis, and 15 had grade 2 degenerative spondylolisthesis. In the Open-TLIF group, the average age of the 24 females and 36 males was 42.2 ± 13.7 years. Forty patients were categorized as grade 1, and twenty were categorized as grade 2. Age, sex, body mass index (BMI), slippage grade, surgical segment, and medical comorbidities did not significantly differ between the two groups **(**Table 1**).** The mean follow-up period for the MIS-TLIF group was 25.15 ± 4.2 months, whereas that for the Open-TLIF group was 24.91 ± 5.7 months.

**Table 1:** Clinical characteristics of studied patients

	Group I (No. = 60) Mean ± SD		Group I (No. = 60) Mean ± SD		T test	*p*.value
Age	40.4 ± 13.6		42.2 ± 13.7		−	0.461
	No	%	No	%	X2	*p*.value
Sex						
Males	34	56.7	34	60.0	0.137	0.711
Females	26	43.3	26	40.0		
Slip grade						
Level 1	29	48.33	26	43.33		
Level 2	31	51.66	34	56.66		
Level						
L4-5	50	83.33	48	80		
L3-4	10	16.66	12	20		
Follow-up period (months)	25.15 ± 4.2		24.91 ± 5.7			
Fusion (modified Bridwell)						
Grade I	40	66.66	35	58.33		0.5602
Grade II	18	33.33	24	41.66		
Complications						
Psudoarthrosis (n = 3)	2	2.4	1	1.8	3.6	0.057
Dural tear (n = 2)	0	0.0	2	100.0	4.0	0.045*
Superficial wound infection (n = 2)	0	0.0	2	100.0	4.0	0.045*
Hospital cost	13,000$		12,500$			0.835

The MIS-TLIF group had significantly longer operation and intraoperative fluoroscopy times than did the Open-TLIF group **(**Table 2**).** However, the MIS-TLIF group exhibited significantly less intraoperative blood loss, postoperative drainage volume, bed rest duration, and hospital stay than did the Open-TLIF group**.**

**Table 2:** Operative data

	Group I (No. = 60) Mean ± SD	Group I (No. = 60) Mean ± SD	T test	*p*.value
Operative time(min)	134.8 ± 26.1	109.7 ± 12.8	6.676	< 0.001**
Estimated blood loss (ml)	256.3 ± 139	550.3 ± 186	− 1.465	0.023*
Irradiation time(sec)	84.4 ± 17.2	29.9 ± 5.4	23.47	< 0.001**
hospital stay (day)	3.2 ± 0.9	6.7 ± 1.6	14.999	< 0.001**

There were no statistically significant differences in the preoperative VAS score for both back and leg pain or in the ODI between the two groups **(**Table 3**).** When comparing each group's postoperative VAS score for back and leg pain and ODI score to their preoperative values at any follow-up time point, substantial improvement was observed **(**Tables 4 and 5**).** The MIS-TLIF group outperformed the Open-TLIF group in terms of the VAS score for back pain and the ODI at less than 6 months following surgery, and the differences were statistically significant. However, at the final follow-up, there were no statistically significant differences in the VAS score for the back between the two groups, but the ODI score was significantly greater in the MIS-TLIF group. Nevertheless, over the course of the follow-up period, the VAS score for leg pain in the mini-open-TLIF group remained better than that in the MIS-TLIF group **(**Table 6**).**

**Table 3:** Comparison between studied groups regarding preoperative VAS back pain, ODI and VAS leg pain

	Group I (No. = 60) Mean ± SD	Group I (No. = 60) Mean ± SD	T test	*p*.value
Preoperative VAS back pain	7.9 ± 0.8	8.1 ± 0.7	− 1.229	0.222
ODI preoperative	48.7 ± 6.6	49.9 ± 6.8	− 0.907	0.366
VAS preoperative leg pain	8.0 ± 0.9	7.7 ± 1.1	1.668	0.098

**Table 4:** Comparison of VAS back pain, ODI and VAS leg pain preoperative, one month, six month and 24 months among group I

	Group I preoperative	1 month	6 months	24 months	F	*p*.value	Partial Eta Squared
VAS back pain	7.9 ± 0.8†‡$	5.6 ± 0.5‡ #$	3.3 ± 0.5#†$	1.6 ± 0.5#†‡	1278.1	< 0.001**	0.96
ODI	48.7 ± 6.6	28.7 ± 4.9	21.5 ± 2.5	21.6 ± 2.3	500.262	< 0.001**	0.895
VAS leg pain	8.0 ± 0.9	4.2 ± 0.7	1.7 ± 0.6	1.6 ± 0.4	1130.241	< 0.001**	0.95

F Value of repeated measures ANOVA

Partial Eta Squared to detect effect size of intervention

^#^ Significance with preoperative

^†^ Significance with1 months postoperative

^‡^ Significance with 6 months postoperative

^$^ Significance with 24 months postoperative

**Table 5:** Comparison of VAS back pain, ODI and VAS leg pain preoperative, one month, six month and 24 months among group II

	Group I preoperative	1 month	6 months	24 months	F	*p*.value	Partial Eta Squared
VAS back pain	8.1 ± 0.7†‡$	6.6 ± 0.4‡ #$	3.5 ± 0.5#†$	1.7 ± 0.7#†‡	1515.9	< 0.001**	0.963
ODI	49.9 ± 6.8†‡$	28.6 ± 4.7‡ #$	24.3 ± 4.2#†$	22.7 ± 2.8#†‡	392.3	< 0.001**	0.869
VAS leg pain	7.7 ± 1.1†‡$	3.9 ± 0.8‡ #$	2.0 ± 0.6#†$	1.4 ± 0.4#†‡	755.8	< 0.001**	0.928

F Value of repeated measures ANOVA

Partial Eta Squared to detect effect size of intervention

^#^Significance with preoperative

^†^ Significance with1 months postoperative

^‡^ Significance with 6 months postoperative

^$^ Significance with 24 months postoperative

**Table 6:** Comparison between studied groups regarding post-operative VAS back pain, ODI and VAS leg pain

		Group I Mean ± SD	Group II Mean ± SD	T test	*p*.value
VAS back pain	After 1 month	5.6 ± 0.5	6.6 ± 0.4	− 10.768	< 0.001**
	After 6 month	3.3 ± 0.5	3.5 ± 0.5	− 1.825	0.071
	After 24 month	1.6 ± 0.5	1.7 ± 0.7	− 0.996	0.321
ODI	After 1 month	21.4 ± 2.4	24.2 ± 4.4	− 4.422	< 0.001**
	After 6 month	21.5 ± 2.5	24.3 ± 4.2	− 4.418	< 0.001**
	After 24 month	21.6 ± 2.3	22.7 ± 2.8	− 2.499	0.014*
VAS leg pain	After 1 month	4.2 ± 0.7	3.9 ± 0.8	2.152	0.033*
	After 6 month	1.7 ± 0.6	2.0 ± 0.6	− 2.206	0.029*
	After 24 month	1.6 ± 0.4	1.4 ± 0.4	2.613	0.01*

When comparing each group's vertebral slippage percentage to its preoperative rank, a statistically significant improvement was observed immediately following surgery until the most recent follow-up. Furthermore, at every follow-up time point, there was no statistically significant difference in the vertebral slip ratio between the MIS-TLIF group and the Open-TLIF group. In both groups, there was a statistically significant difference in the segmental lordosis of the slipping level and the lumbar lordosis between the pre- and postoperative periods. However, when comparing preoperation, immediately postoperatively, and segmental lordosis, no discernible changes were detected between the two groups. On the other hand, there was more segmental lumbar lordosis in the Open-TLIF group. The postoperative interbody fusion rate did not significantly differ between the two groups according to the Bridwell classification (*p* < 0.05) (Table 7).

**Table 7:** Comparison between studied groups regarding radiological outcome

	MIS-TLIF	Mini-Open TLIF
Vertebral slip %		
Pre-operative	24.23 ± 4.22	25.33 ± 5.33‡
Post-operative	8.67 ± 2.91*	8.45 ± 2.55*‡
Last follow up	8.99 ± 2.3*†	8.87 ± 2.1*†‡
Lumbar lordosis		
Pre-operative	43.3 ± 7.9	42.1 ± 8.5‡
Post-operative	48.1 ± 8.2*	48.7 ± 7.6*‡
Last follow up	47.2 ± 7.9*†	48.5 ± 6.2*†‡
Focal lordosis		
Pre-operative	5.4 ± 5.9	6.5 ± 4.3‡
Post-operative	8.9 ± 7.8*	9.2 ± 5.1*‡
Last follow up	8.1 ± 6.7*†	8.2 ± 4.9*†‡

Vertebral slip percentage, Lumbar lordosis and focal lordosis comparison between the two groups: *P < 0.05, comparing post-operative data in each group compared to preoperative data, † *P* > 0.05, comparing data at last follow-up versus immediately following surgery. ‡ *P* > 0.05, comparing the two groups'

There were two occurrences of superficial incision infection in the open-TLIF group and none in the MIS-TLIF group. Anti-infection medications and frequent dressing changes were used to treat both infections. Two incidences of intraoperative dural tears were recorded in the mini-open-TLIF group; these were immediately repaired, and there was no postoperative leakage of CSF. With two cases of nonunion, the MIS group had a greater rate of pseudoarthrosis. Patients, however, declined to have the procedure repeated.

## Discussion

Numerous published lumbar interbody fusion techniques have been developed with the goal of maintaining spinal alignment, boosting fusion rates, and reducing back and leg discomfort. To achieve smaller surgical wounds, less trauma to surrounding tissue, and a quicker recovery after surgery, Foley et al. introduced MIS-TLIF as an alternative to conventional open-TLIF in the early 2000s. However, similar long-term results for both MIS- and open-TLIF have been previously described in the literature. On the other hand, the positive impacts of MIS-TLIF might be evident in the early stages of recovery, with a shorter period of postoperative opioid use, a shorter hospital stay and an earlier return to work following surgery [15]. However, strong data are currently lacking on which technique is more clinically effective in treating symptomatic low-grade degenerative spondylolisthesis. This study aimed to compare and evaluate the safety and effectiveness of mini-open-TLIF and MIS-TLIF in the treatment of degenerative spondylolisthesis. To prevent bias, the same surgical team with extensive training in the MIS/mini Open-TLIF approach handled every patient in our study.

The paraspinal muscles sustain more damage when they are separated from their origin/insertion during the mini-open-TLIF. On the other hand, MIS-TLIF involves the use of the paraspinal approach, which aims to dilate the muscles to minimize muscular damage. This was observed in our study because the amount of blood loss was greater in the mini-open-TLIF group. Evaniew et al. [3] reported that patients who underwent MIS experienced reduced blood loss and quicker surgery. Similar to the findings of a previous study, Qin et al. [16] demonstrated that MIS-TLIFs reduce blood loss better than do mini-open TLIFs; nevertheless, the authors noted that the MIS-TLIF group needed more time during surgery. Similar outcomes to our investigation were observed in the previous trial, with the MIS group requiring more time during surgery and less blood loss. This could be attributed to the surgical and technique learning curve and familiarity with the approach with fewer bony landmarks and working in a narrow tube.

It is important to consider the cumulative effects of radiation exposure on both the patient and the surgical team [7]. The smaller operating field, difficulty in seeing bone landmarks, and challenging learning curve associated with MIS-TLIF all contribute to greater radiation exposure duration. According to our study, the prolonged fluoroscopy time needed for percutaneous pedicle screw implantation with MISTLIF accounts for the majority of the increased radiation exposure compared to that of mini-open-TLIF. The radiation exposure period could be shortened with more surgical experience and an improvement in the learning curve, as we noticed in the latter patients who underwent MIS-TLIF [14].

In the current economic environment, comparing the cost-effectiveness of MIS-TLIF versus mini-open-TLIF is crucial. In two papers published in 2012 and 2014, Parker et al. computed ICERs taking into account both direct and indirect costs; however, the results were inconsistent. A statistically significant difference in cost between the two procedures was not found by our study's cost comparison. However, MIS-TLIF implants were more costly than Open-TLIF implants. This may be explained by the fact that the open-TLIF group required a longer postoperative hospital stay (6.7 ± 1.6 days) together with the indirect cost of delayed return to work in comparison to 3.2 ± 0.9 days in the MIS group. Early hospital discharge minimizes exposure to nosocomial infections, promotes earlier and more frequent mobility, and decreases hospital expenses.

Both groups' back and leg pain VAS scores and ODI scores significantly improved following surgery compared to the preoperative values. This demonstrates that both approaches have proven successful in addressing those suffering from degenerative spondylolisthesis, as reported in the literature [1, 7, 16].

Furthermore, when treating degenerative lumbar illnesses, Heemserk et al. [8] demonstrated that MIS and open operations have comparable outcomes at two years of follow-up. Qin et al. [16] reported that when treating single-level low-grade spondylolisthesis, MIS-TLIF seems to be a more effective and safer approach and has a better long-term functional prognosis. The sole difference in the quality-of-life score between the two groups in our study was discovered during follow-up, as the MIS-TLIF group outperformed the Open-TLIF group during the first month in terms of the VAS score for back pain and during the first 6 months in terms of the ODI score for disability (*p* < 0.001). The open-TLIF group, however, had a greater VAS score for leg pain relief (*p* < 0.05) than did the MIS group. This suggests that although the two groups' clinical performance was similar over time, the MIS-TLIF group performed better than the Open-TLIF group only in terms of early reduction in low back pain and disability. However, Open-TLIF is better for neural tissue decompression because of the large exposure of the approach.

Currently, vertebral slip reduction in spondylolisthesis patients remains a controversial topic. Several studies have shown that satisfaction rates ranging from 75 to 80% can be achieved with in situ fusion without reducing slippage [4, 13]. Conversely, other studies have reported a notable prevalence of sagittal imbalance, progressive slippage and pseudoarthrosis. Reducing degenerative spondylolisthesis will somewhat enhance the sagittal lumbosacral balance and the clinical outcome even if it is not required and provide areas for bony fusion [4]. In our study, both techniques showed significant changes in the slip degree, with no difference between the two techniques. The discrepancy in the reduction data may be limited to patients with high-grade spondylolisthesis, and all our patients had low-grade spondylolisthesis [1].

Higher grades of spondylolisthesis are characterized by a sagittal imbalance caused by slippage and lordosis loss (LL), which are correlated with disc degeneration. Pelvic retroversion, the result of the pelvis rotating, causes the sacral slope (SS) to decrease and the pelvic tilt (PT) to increase, which restricts the anterior translation of the axis of gravity. Therefore, the restoration of the normal PT range requires the restoration of lumbar lordosis [1, 17]. Lower back discomfort can arise from relative kyphosis on the fused segment caused by a decrease in postoperative LL. This, in turn, increases the tensile stress on the spinal structures posterior to the fused segment, including the paraspinal muscles. Therefore, the restoration of segmental and total lordosis is essential for these patients.

Compared to MIS-TLIF, a few surgeons have theorized that open TLIF would allow for more segmental and global lordosis correction. After MIS-TLIF, Dibble et al. [2] observed greater improvements in segmental lordosis (SL). However, there were no variations in the global lordosis angle compared to that of the Open-TLIF group. On the other hand, the global lordosis angle did not vary. We discovered the same findings during our investigation, with no statistically significant difference in segmental or global lordosis between the two groups. On the other hand, LL and SL significantly changed between the preoperative and postoperative data.

The postoperative interbody fusion rate did not significantly differ between the two groups according to the Bridwell classification (p < 0.05). This finding implies that the fusion rate was unaffected by the surgical technique. In a 5-year follow-up investigation on the fusion findings of MIS-TLIF, Kim et al. [9], in a narrative review of 14 prospective observational studies and 6 randomized controlled trials, estimated a fusion rate of more than 90%. This is consistent with the results of our study. It could be concluded that for both mini-opening and MIS, the surgical fusion rate may produce good outcomes. However, a number of studies comparing the two methods of treatment have suggested that open-TLIF may provide superior interbody space preparation compared with MIS-TLIF and that MIS-TLIF may result in a lower fusion rate than open-TLIF [9, 14]. Many other factors may contribute to this discrepancy, such as the type of graft used (either autograft or synthetic). Sufficient autografts were used in all patients in this study.

According to Goertz et al. [6] and Krüger M.T. et al. [10], obese patients who underwent MIS-TLF surgery had a greater incidence of dural tears. The use of TLIF through tube retractors placed directly over the facet avoids the need for retraction of the dura, which avoids the incidence of tears and the avoidance of dural tears during the insertion of the roods or the set esrew at the final step in Open-TLIF. The open-TLIF group experienced an intraoperative dural tear in two patients; however, the MIS-TLIF group experienced no such problems. The use of skin retractors resulted in two cases of skin edge necrosis in the Open-TLIF group, which were further worsened by superficial skin infections. Using a tubular retractor prevented complications in the MIS-TLIF group by preventing significant skin retraction and causing less tissue injury, which minimized the infection rate.

## Limitations

There are several limitations to this study. First, the study was a single-center retrospective study and included only a small number of scenarios. Second, the duration of patient follow-up was somewhat brief. Third, the analysis included only patients who had single-level low-grade spondylolisthesis, and all of them had disc-level lesions at the L4-L5 and L5-S1 levels. More research is required on people with higher-level spondylolisthesis and multisegment disc degenerative diseases.

## Conclusion

MIS-TLIF and mini-open-TLIF can be surgically effective in treating single-level degenerative lumbar spine spondylolisthesis in properly selected patients. More specifically, MIS-TLIF can significantly reduce bleeding and the length of hospital stay; nevertheless, MIS-TLIF is associated with increased radiological exposure.

## Data Availability

No datasets were generated or analysed during the current study.
